# Bridging knowledge gaps: An observational study on HPV awareness and misconceptions among young adults in China

**DOI:** 10.1371/journal.pone.0337518

**Published:** 2025-12-01

**Authors:** Minrui Li, Lili Liang, Xuanyan Chen, Zhoujun Zhu, Chenyan Fang, Runan Zhou

**Affiliations:** 1 Guangdong University of Finance and Economics, School of Humanities and Communication, Guangzhou, China; 2 Department for Gynecology, HPV Research Lab, Charité-Universitätsmedizin Berlin, Corporate Member of Freie Universität Berlin and Humboldt-Universität zu Berlin, Berlin, Germany; 3 Sun Yat-sen University, School of Journalism and Communication, Guangzhou, China; 4 Department of Gynecologic Oncology, Zhejiang Cancer Hospital, Hangzhou, Zhejiang, China; Federal University Otuoke, NIGERIA

## Abstract

Human Papillomavirus (HPV) is a sexually transmitted infection associated with genital warts and multiple cancers. HPV awareness and vaccine coverage remain low in mainland China. With the rise of digital social media, young people increasingly acquire knowledge and information online, influencing their perceptions and decisions. Impact of social media on HPV knowledge and vaccine hesitancy remains unclear. The study aimed to assess the knowledge and awareness of HPV and its vaccines hesitancy among young adults in China using an online structured questionnaire with implied consent. Our findings showed that 94.4% of respondents had heard of HPV, with most aware of its sexual transmission and risks to both men and women. However, gaps in knowledge and misconceptions were identified, particularly concerning the asymptomatic nature of HPV infections and the lack of effective antiviral treatments for HPV. Misconceptions about HPV and vaccine safety were notable, contributing to vaccine hesitancy. Yet 78.4% of participants believed in the effectiveness of HPV vaccine, and 83.5% expressed their willingness to receive vaccination if recommended by a doctor. Although overall awareness of HPV and HPV vaccination was high, significant knowledge gaps and misconceptions remain. Thereby reshaping public perception and enhancing awareness, trust and motivation for HPV vaccination are under challenge. Addressing misinformation through strategic educational initiatives could enhance trust in HPV vaccination and improve coverage, ultimately reducing the burden of HPV-related diseases in China.

## 1 Introduction

Human Papillomaviruses (HPVs) are a diverse group of small DNA viruses, comprising approximately 450 different subtypes, that primarily infect the skin and mucosal surfaces [1]. The incidence of HPV is particularly high among young adults, peaking around 25–29 years of age before stabilizing or slightly declining [2]. HPV infection is the most common viral infection worldwide, with many cases being subclinical or asymptomatic infections [1]. Often the individual might not notice the infection unless an HPV genotyping test was done. While some HPV types, notably HPV6 and 11, cause benign lesions such as genital warts and papilloma [1,3], which are classified as sexually transmitted infected diseases, others are associated with more severe outcomes. Persistent infection with high-risk HPV types, particularly HPV 16 and 18, can lead to pre-malignant dysplasia that may progress into HPV-associated cancers of cervix, vagina, vulva, anus, penis, and oropharynx [3]. Indeed, hrHPVs are detected in over 90% of cervical cancer cases [4], underscoring their significant public health impact around the world [3].

The development of prophylactic vaccines targeting the most relevant HPV subtypes has proven highly effective in preventing HPV infections and their associated cancers [3]. These vaccines are available in bivalent (against HPV 16 and 18), quadrivalent (against HPV 6, 11, 16 and 18) and 9-valent (against HPV 6, 11, 16, 18, 31, 33, 45, 52 and 58) [5]. Since the first global launch of an HPV vaccine in 2006, Chinese authorities eventually approved Cervarix (GlaxoSmithKline Biologicals SA, Belgium) in 2016, Gardasil 4 (Merck & Co., USA) in 2017, and Gardasil 9 (Merck & Co., Inc) in 2018 for use in mainland China [6,7]. To alleviate the global burden of HPV-related diseases, the World Health Organization (WHO) has set an ambitious goal that 90% girls aged 9–14 years to be fully vaccinated against HPV by 2030 [8]. Despite the global initiative, China bears the second-highest absolute number of cervical cancer cases and deaths worldwide [5,9]. According to the WHO IARC GLOBOCAN 2022 estimates, the age-standardized incidence rate in China is approximately 13.8 per 100,000 women, and the mortality rate is 4.5 per 100,000. While the absolute numbers of cases and deaths are among the highest globally, the rates are relatively lower than in some other regions, largely due to its large population base [5,10,11]. The financial burden is substantial; a study by Chen et al. estimated the average cost of invasive cervical cancer in China ranging from US Dollar ($) 15,034.9 to $ 27,332.6 depending on disease stage [12], imposing a significant economic impact on families and the healthcare system. Similar economic impacts have been documented in other countries, highlighting the universal cost of HPV-related morbidity.

Recent data report relatively low HPV vaccination coverage across mainland China, with stark disparities among different age groups [9]. The highest first-dose HPV vaccine coverage (14.2%) was observed among women aged 20–24 years, while the lowest was in the 9–14 age group (4.0%) [9]. Full three-dose coverage was similarly uneven, with the highest rate in the 25–29 age group (9.39%) and the lowest in the 9–14 age group (0.31%) in 2022 [9]. These disparities are thought to result from a range of factors, including limited public knowledge, regional health care access, vaccine cost, and gender-specific policy limitations [6]. Financial issues might be a major consideration for vaccine-uptake in mainland China. Even if manufacturers adopt a low-price strategy, creating a more affordable and accessible vaccine environment, compared to imported vaccine (ranging from $ 299-$597 in Hong Kong, China), the price of domestic vaccine may cost less than $150, which is still the most expensive vaccine in mainland China [6]. Yet despite the expanded availability and reduced cost, vaccine uptake remains low. This evolving scenario suggests the importance of addressing persistent gaps in public awareness and combating vaccine hesitancy driven by misinformation.

Understanding the attitudes of younger Chinese adults-especially those Z-generation are individuals born around the turn of the 21st century-is key to identify new intervention opportunities. These demographic has been raised and grown up in a digital connected society, with broad access to education and online information, including health content disseminated through video-based and interactive social media platforms, representing a group with potentially high levels of HPV awareness [13]. However, HPV coverage among young girls is alarmingly low [9]. Existing studies have initially focused on public awareness or older generation, leaving a gap in understanding the specific attitudes and knowledge of the younger generation, especially those who belong to tech-savvy cohorts. In recent years, digital media and social networking platforms have emerged as powerful tools in public health education. Platforms like *Zhihu, Weibo, TikTok, WeChat,* and *Xiaohongshu (RED)* are widely used by younger Chinese populations and have shown protentional in disseminating HPV-related content. Studies have reported that social-media based interventions can improve health related knowledge, correct misinformation, and increase HPV -related awareness [14,15]. However, few have specifically focused on the Z-generation in China- an emerging demographic with high digital literacy but low vaccine coverage. Health Belief Model (HBM) is one of the most widely used conceptual frameworks under cognitive theory and developed to explain why many people fail to participate in programs preventing disease [16–18]. In this study we applied the HBM with adjustments to evaluate the health behaviour and to explain changes in HPV associated health behaviour.

The present study aims to explore the level of knowledge, awareness, and attitudes towards HPV and HPV vaccination among Chinese young adults, the Z-generation. By focusing on this cohort, we designed and conducted the current study to provide valuable insights into the current state of HPV awareness among young, educated Chinese adults and highlight critical areas where public health initiatives can be strengthened. Additionally, awareness levels still vary widely across different regions and populations, often reflecting disparities in economic and educational access. By examining the influence of social media on health information dissemination, this research also aims to inform the development of more effective, targeted communication strategies to improve HPV vaccination coverage in China.

## 2 Materials and methods

### 2.1 Study design, ethical approval and participants

The observational study received approval from the institutional review board (IRB) of Sun Yat-sen University (SJC-IRB-2024004) on April 25, 2024, and was conducted in the accordance with institutional, national and international ethical guidelines, including the principles of the 1964 Helsinki Declaration and its later amendments. Neither participants, the funders, nor the public were involved in the study design, conduct, data collection and analysis, decision to publish, or preparation of the manuscript.

Informed consent was implied through the voluntary completion of the anonymous online questionnaire, as approved by the IRB. Before accessing the survey, participants were provided with an online information sheet detailing the purpose of the study, its voluntary nature, the anonymity and confidentiality of their responses. They were informed of their right to withdraw at any time without explanation. Only those who agreed to participate proceeded with the survey.

All participants were 18 years or older. Between 26.04.2024 and 22.05.2024, a total of 250 male and female participants were recruited using convenience sampling from Sun Yat-sen University. Research team members approached eligible students on campus and via university-affiliated online channels, such as WeChat student groups. The number of students approached was not systematically recorded, and therefore, cannot calculate a precise response rate. Findings should be interpreted as representing the responses of this specific student group, rather than the broader university or general population. The sample size was calculated using the formular for estimating a single proportion. Assuming a conservative proportion (p) of 0.5 for maximum variability, a 95% confidence level, and a margin of error (d) of 0.07, a minimum samples size of 196 participants was required. Our final sample of 250 participants exceeded this requirement.

Data collection was conducted online using a professional online questionnaire survey platform, miniApp called *Wenjuanxing (Zhongyan Technology Co., Ltd. Changsha, China)*, easily accessible via phone, tablet, or computer. No compensation or financial reward was provided for participation.

### 2.2 Questionnaire content

The questionnaire was available in both English and Chinese (S1 Appendix_EN and S2 Appendix_CN) and underwent content validation and piloting. Three medical doctors validated the content for relevance and accuracy. A piloted test with 50 conveniently selected participants was conducted to evaluate clarity and readability, resulting in a Cronbach’s alpha value of 0.816, indicating good internal consistency and reliability. Nevertheless, we acknowledge that some items-particularly those related to subjective concepts such as frequency or severity-may have been interpreted differently by respondents. The raw data from surveys is provided in S3 Dataset as Supporting Information.

The survey consists of four key perspectives: 1) demographic data; 2) basic knowledge of HPV and HPV infection; 3) health beliefs about HPV infection; and 4) concerns and attitudes about HPV vaccination. In the section assessing basic knowledge of HPV and HPV infection, participants responded to questions with ‘yes’, ‘no’, and ‘don’t know/not sure’. Correct answers were not indicated to participants, and the correct rate (%) was later analysed to assess general awareness rather than factual knowledge in all cases.

The HBM was selected as the conceptual framework for this study as it effectively captures the psychosocial factors influencing health behaviours like vaccination [16–18]. While other validated HPV knowledge and assess perceptions that directly predict behavioural intention, such as perceived susceptibility, benefits, and barriers. This approach aligns with recent studies investigating HPV vaccine hesitancy in similar young adult population [16,17]. This section comprised 18 items categorized into five domains: 1) perceived susceptibility to HPV infection (items 1–4); 2) perceived severity of potential HPV infection (items 5–8); 3) perceived benefits of preventing HPV infection (items 9–12); 4) potential barriers to HPV vaccination (items 13–15); and 5) self-efficacy related to receiving HPV vaccination (items 16–18). Participants rated their agreement with each statement using a 5-point Likert scale, ranging from ‘Strongly disagree’ to ‘Strongly agree’. Mean scale scores for each item were calculated and compared with corrected Likert-intervals as suggested by J. Pimentel [19], to predict the willingness of participants to receive HPV vaccine. Pimentel intervals are designed to produce more accurate confidence intervals for proportions by accounting for the ordinal nature of Likert scale data. Unlike standard methods, they avoid assuming equal distances between response options, helping to prevent overinterpretation of numerical values and providing more reliable estimates of variability [19,20]. The corrected Likert-intervals were applied to represent the five response categories: strongly disagree (interval 1.00–1.79), disagree (interval 1.80–2.56), neutral (interval 2.60–3.39), agree (interval 3.40–4.19), and strongly agree (interval 4.20–5.00).

## 3 Statistical analysis

All data were entered and analysed via SPSS software, Version 23. Descriptive statistics were used to analyse demographic data, and to assess overall awareness and misconceptions. Categorical variables were summarized as frequencies and percentages. Bivariate analyses were conducted to test specific hypotheses: 1) that there would be no significant differences in concerns about the HPV vaccine (side effects, cost, safety) between males and females (using χ² Test, as presented in Table 4); and 2) that there would be a significant association between gender and trust in social media as a source of HPV information (using χ² Test, as presented in Table 5). In the HBM section, a mean value was calculated for each item using the Likert scale. The distribution of mean scale scores across the corrected Likert-intervals was then used to assess participants’ perceptions and predict their willingness to receive the HPV vaccine. As the online questionnaire required responses to all fields before submission, there were no missing data, and thus no imputation methods were required.

**Table 4:** Greatest concern(s) for HPV vaccination by sex.

Items	In total, n (%)	Female, n (%)	Male, n (%)	p-value*
Are potential side effects your greatest concern about HPV vaccine?	187 (79.2)	119 (78.8)	68 (80.0)	0.828
Is cost your greatest concern about HPV vaccine?	152 (64.4)	102 (67.5)	50 (58.8)	0.179
Is the effectiveness and safety of the vaccine your greatest concern about HPV vaccine?	41 (1.58)	26 (17.2)	15 (17.6)	0.934
Is any other concern not listed here your greatest concern about HPV vaccine?	10 (4.2)	9 (6.0)	1 (1.2)	0.080
**Cases in total, n (%)**	236 (100)	151 (64.0)	85 (36.0)	

Abbreviation: HPV = Human Papillomavirus. * p-value were calculated by χ² test comparing responses between females and males. No statistically significant differences (p < 0.05) were observed across any concern categories.

**Table 5:** Sex Differences in Sourcing and Trust of Social Media for HPV Information.

Characteristic	In total, n (%)	Female, n (%)	Male, n (%)	P-value*
**HPV knowledge from social media**				
yes	183 (77.5)	121 (51.3)	62 (26.3)	0.260
no	53 (22.5)	30 (12.7)	23 (9.7)	
**How do you trust the social media**				
trustful	98 (41.5)	66 (28.0)	32 (13.6)	0.034
Neutral	116 (49.2)	76 (32.2)	40 (16.9)	
Untrustful	23 (9.3)	9 (3.8)	14 (5.9)	

* p-value were calculated by χ² test comparing differences between females and males. Statistically significant differences were observed across any concern categories if p < 0.05.

## 4 Results

A total of 250 participants completed the study. The demographic characteristics of the attendees are summarized in Table 1. Among the respondents, 62.0% (n = 155/250) were female, and 85.2% were aged 18–26 years. The majority (94.0%) belonged to the Han ethnic group, and 89.6% were majoring in non-biomedical field, while 10.4% were in biomedical-related majors. No statistically significant difference in educational level was observed between females and males (*p* = 0.584, χ²-Test), indicating that the educational differences between genders did not influence the respondents’ awareness or concerns.

**Table 1:** Demographic characteristics.

Variables	N (%)
Gender	
Female	155 (62.0)
Male	95 (38.0)
Age, years	
18-26	213 (85.2)
> 26	37 (14.8)
Ethnic groups*	
Han	235 (94.0)
others	15 (6.0)
Educational level	
Junior college	210 (84.0)
postgraduate	15 (16.0)
Major in University	
Biomedical related	26 (10.4)
Non-biomedical related	224 (89.6)
In total	250 (100)

* In addition to Han, other ethnic groups in the study included Zhuang, Hui, Miao, Manchu, Yi, Tujia, Mongols, Dong, Bai, and Shui (listed by population).

### 4.1 Basic knowledge and awareness of HPV and HPV vaccination

To assess the understanding of HPV and HPV vaccination, participants’ basic knowledge and awareness were weighed up using 18 statements (Table 2). Of the 236 participants aware of HPV, knowledge was high regarding its sexual transmission (63.6%, n = 150/236), its ability to infect both men and women (78.0%, n = 184/236), and its link to unsafe sex (83.1%, n = 196/236). Over 60% understood its link to cervical cancer and other cancers. Most believed in the vaccine’s efficacy (78.4%, n = 185/236) and worth (81.8%, n = 193/236). However, critical knowledge gaps were identified: only 28.8% (n = 68/236) knew HPV infections are often asymptomatic, and merely 12.7% (n = 30/236) were aware there is no antiviral treatment for the infection itself. Common misconceptions included beliefs that the vaccine affects the menstrual cycle (37.7% correct response rate) and that it is too late to vaccinate after infection (36.0% correct response rate).

**Table 2:** Basic knowledge and awareness of HPV and HPV vaccination.

Items	Correct, n (%)	Incorrect, n (%)	Don’t know/not sure, n (%)
HPV is a sexually transmitted virus.	150 (63.6)	46 (19.5)	40 (16.9)
HPV is very common in China.	151 (64.0)	34 (14.4)	51 (21.6)
Both men and women are susceptible to HPV.	184 (78.0)	17 (7.2)	35 (14.8)
Regardless of sexual orientation, having unsafe sexual activity increase the risk of HPV infection.	196 (83.1)	13 (5.5)	27 (11.4)
Most people who get HPV infection do not have any symptoms.	68 (28.8)	79 (33.5)	89 (37.7)
HPV can lead to benign genital warts.	135 (57.2)	24 (10.2)	77 (32.6)
Persistent HPV infection in women can lead to abnormal cervical dysplasia and/or cervical cancer.	146 (61.9)	20 (8.4)	70 (29.7)
HPV can cause cancers of the oropharynx, penis, and anus in both men and women.	145 (61.4)	17 (7.2)	74 (31.4)
There is no treatment for HPV infection.	30 (12.7)	142 (60.2)	64 (27.1)
There is treatment for HPV induced warts.	108 (45.8)	40 (16.9)	88 (37.3)
HPV is associated with infertility.	139 (58.9)	19 (8.0)	78 (33.1)
HPV vaccine is effective.	185 (78.4)	10 (4.2)	41 (17.4)
It is worth getting HPV vaccine.	193 (81.8)	11 (4.6)	32 (13.6)
Vaccination against HPV will prevent HPV-driven cancers, not just cervical cancer.	186 (78.8)	7 (3.0)	43 (18.2)
HPV vaccines can adversely affect the immune system.	67 (28.4)	76 (32.2)	93 (39.4)
HPV vaccines affect menstrual cycle.	89 (37.7)	56 (23.7)	91 (38.6)
Men should also be vaccinated against HPV.	193 (81.8)	34 (14.4)	9 (3.8)
It is too late to get the HPV vaccine if the individual has got HPV infected.	85 (36.0)	75 (31.8)	76 (32.2)
**In total**			n = 236

Abbreviation: HPV = Human Papillomavirus.

### 4.2 Health belief about HPV and HPV vaccination

**Fig 1:**
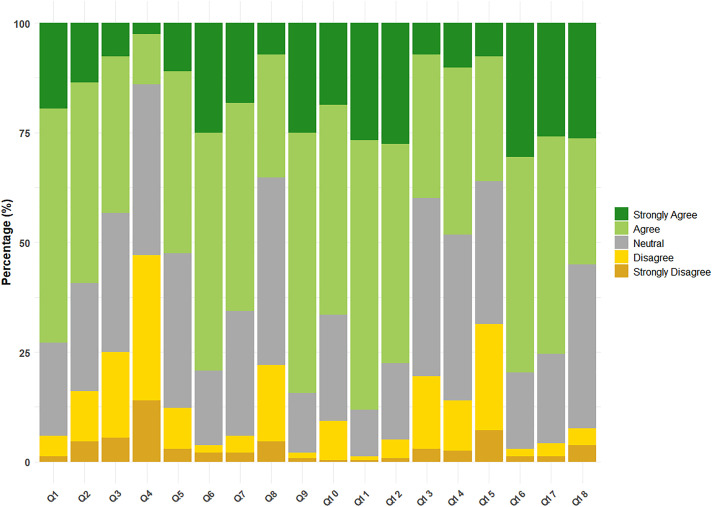
Stacked bar charts showing respondents’ health beliefs about HPV and the HPV vaccines (items Q1-Q18), using 5-point (5’) Likert Scale (5’ = strongly agree to 1’ = strongly disagree). Bars indicate the percentage of responses in each category, color-coded as: strongly agree (deep green), agree (light green), neutral (grey), disagree (yellow), and strongly disagree (brown). Number Q1 to Q18 in the X axis indicates the 18 different items in the health belief model.

The HBM was further employed to predict participants’ willingness to receive HPV vaccine. Responses were measured on a 5-point (5’) Likert scale (1’ = strongly disagree, 5’ = strongly agree) and are presented in Table 3 and visualized in Fig 1. While Table 3 shows mean scale scores for each item, indicating the overall attitude and interpreted through five corrected Likert-intervals, the stacked bar charts in Fig 1 display the percentage of responses in each category, providing a detailed view of the response distribution.

**Table 3:** Health belief toward HPV and the HPV vaccines.

Nr.	Items	5-point (5’) Likert Scale *, n (%)					Mean Scale**
		5’	4’	3’	2’	1’	
Q1	Anyone who is sexually active is at risk of HPV infection.	46 (19.5)	126 (53.4)	50 (21.2)	11 (4.7)	3 (1.3)	**3.85**
Q2	Many people can be infected with HPV, including my relatives, partners, and friends.	32 (13.6)	108 (45.8)	58 (24.6)	27 (11.4)	11 (4.7)	**3.52**
Q3	I may be infected with HPV in the future.	18 (7.6)	84 (35.6)	75 (31.8)	46 (19.5)	13 (5.5)	**3.20**
Q4	I am at high risk for HPV infection.	6 (2.5)	27 (11.4)	92 (39.0)	78 (33.1)	33 (14.0)	**2.56**
Q5	HPV infection is terrible.	26 (11.0)	98 (41.5)	83 (35.2)	22 (9.3)	7 (3.0)	**3.48**
Q6	HPV infection can lead to serious health problem, threatening your wellbeing.	59 (25.0)	128 (54.2)	40 (16.9)	4 (1.7)	5 (2.1)	**3.98**
Q7	HPV infection can seriously affect your daily life.	43 (18.2)	112 (47.5)	67 (28.4)	9 (3.8)	5 (2.1)	**3.76**
Q8	HPV infection can cause death.	17 (7.2)	66 (28.0)	101 (42.8)	41 (17.4)	11 (4.7)	**3.16**
Q9	HPV vaccination can benefit me.	59 (25.0)	140 (59.3)	32 (13.6)	3 (1.3)	2 (0.8)	**4.06**
Q10	HPV vaccination can help my immune system fighting against HPV.	44 (18.6)	113 (47.9)	57 (24.2)	21 (8.9)	1 (0.4)	**3.75**
Q11	HPV vaccination can reduce the risk of HPV infection.	63 (26.7)	145 (61.4)	25 (10.6)	2 (0.8)	1 (0.4)	**4.13**
Q12	HPV vaccination can make sexual activity safer.	65 (27.5)	118 (50.0)	41 (17.4)	10 (4.2)	2 (0.8)	**3.99**
Q13	I am concerned about the safety of the HPV vaccine.	17 (7.2)	77 (32.6)	96 (40.7)	39 (16.5)	7 (3.0)	**3.25**
Q14	I am concerned about the potential side effects of the HPV vaccine.	24 (10.2)	90 (38.1)	89 (37.7)	27 (11.4)	6 (2.5)	**3.42**
Q15	I am worried that getting the HPV vaccine will take up too much of my time and effort.	18 (7.6)	67 (28.4)	77 (32.6)	57 (24.2)	17 (7.2)	**3.05**
Q16	I would consider getting the HPV vaccine.	72 (30.5)	116 (49.2)	41 (17.4)	4 (1.7)	3 (1.3)	**4.06**
Q17	I am going to get the HPV vaccine.	61 (25.8)	117 (49.6)	48 (20.3)	7 (3.0)	3 (1.3)	**3.96**
Q18	I will get the HPV vaccine.	62 (26.3)	68 (28.8)	88 (37.3)	9 (3.8)	9 (3.8)	**3.70**

* The 5-point (5’) Likert Scale indicates: 5’ = strongly agree; 4’ = agree; 3’ = neutral; 2’ = disagree; 1’ = strongly disagree. ** The mean scale scores the overall attitude of the responders towards each item. Mean scale for each item was calculated and interpreted into five response categories through corrected Likert-intervals: strongly disagree (interval 1.00–1.79), disagree (interval 1.80–2.56), neutral (interval 2.60–3.39), agree (interval 3.40–4.19), and strongly agree (interval 4.20–5.00).

Respondents perceived HPV as highly infectious to the general population (mean scale 3.52), especially among sexually active individuals (mean scale 3.85). However, they perceived their own susceptibility as lower (mean scale 2.56–3.20).

Higher mean scale (3.48–3.98) were noted in assessing the perceived severity of potential HPV infection, with the belief that ‘HPV infection can cause death’ scoring 3.16. Attendees expressed strong confidence in the benefits of HPV vaccination and motivation for vaccination in its effectiveness with mean scale ranging from 3.75 to 4.13. However, concerns about the vaccine’s safety and potential side effects remained (mean scale 3.25–3.42), as did worries about the time and effort required for HPV vaccination (mean scale 3.05). Overall, participants showed a positive attitude towards HPV vaccine uptake (mean scale 3.70–4.06). At last, most of the respondents (83.5%, n = 197/236) indicated that they would be willing to receive the HPV vaccine if recommended by a doctor, while only 4.7% (n = 11/236) stated they would decline such a recommendation.

### 4.3 Concerns about HPV vaccine

To further explore concerns and attitudes about HPV vaccine, additional questions were analyzed by sex and overall, as shown in Table 4. The most common concern was the potential side effects of HPV vaccine, cited by 79.2% (n = 187/236) of respondents. Cost of the HPV vaccine was also a significant concern, with 64.4% (n = 152/236) expressing their worry. In contrast, only 1.58% (n = 41/236) of respondents doubted the effectiveness and safety of the vaccine. Concerns about HPV vaccine side effects were similar between females (78.8%) and males (80.0%) (p = 0.828). No significant sex-based differences were observed for cost (p = 0.179), vaccine effectiveness/safety (p = 0.934), or other concerns (p = 0.080).

### 4.4 Sex differences in sourcing and trust of social media for HPV information

To investigate the role of social media as a source for HPV information dissemination, participants were asked about their usage and trust. Results were showed in Table 5. Of the participants who had heard of HPV or the HPV vaccines, most (77.5%, n = 183/236) reported obtaining related knowledge from social media with no statistical differences observed between female and male participants (p = 0.260), highlighting the powerful influence of social media on health information dissemination. A notable sex difference was observed in social media usage, with a higher proportion of female participants (51.3%, n = 121/236) reporting obtaining information through social media compared to males (26.3%, n = 62/236).

Regarding trust in the accuracy of HPV related information obtained on social media, opinions were mixed: 41.5% (n = 98/236) regarded it as trustworthy, while 49.2% (n = 116/236) were neutral. However, a statistically significant association was found between sex and trust (p = 0.034). This association was primarily driven by distrust; although only 9.3% (n = 23/236) of all participants found social media untrustworthy, male respondents were disproportionately represented in this group.

## 5 Discussion

This study assessed HPV knowledge, misconceptions, and health beliefs among young adults in China. Overall, a high awareness of HPV (94.4%) and strong belief in vaccine efficacy was found. However, substantial gaps in understanding the infection’s nature history and prevalent misconceptions about vaccine safety were identified. Although participants generally perceived HPV as a severe disease, many underestimated their personal susceptibility. Social media was the primary information source for most (77.5%), but trust in this information varies significantly by gender.

Our finding of a high overall awareness is consistent with recent studies among university students in China [21], confirming a positive trend in HPV knowledge among educated youth. Nevertheless, specific misconceptions remain, particularly regarding the asymptomatic nature of HPV and the misconception that vaccination is irrelevant after infection. These recurring patterns highlight a common and ongoing challenge that extends beyond our sample [21]. For example, our data reveal that a striking 31.8% of respondents incorrectly believed that vaccination is ineffective after HPV infection and another 32.2% expressed uncertainty. Despite these misconceptions and knowledge gaps, the majority held positive attitudes towards the vaccine: 78.4% trusted the effectiveness of HPV vaccine, and 81.8% believed that receiving HPV vaccine was worthwhile. Unique to our study is the detailed application of the HBM, which revealed a disconnect between the high perceived severity of HPV-related diseases (mean scale 3.16–3.98) and the neutral perception of personal susceptibility (mean scale 2.56–3.20). This nuance might be missed in knowledge-based surveys. The HBM assessment also showed a strong self-efficacy regarding vaccination (mean scale 3.70–4.06), consistent with prior reports [21–24], while perceived barriers to vaccination such as safety concerns were moderate (mean scale 3.05–3.42).

The financial burden of vaccination emerged as a major barrier in our study and is well-documented in the Chinese context [5,8], particularly among students without a regular income. This emphasizes the importance of recent policy developments, notably the Chinese government’s announcement to incorporate HPV vaccination into the national immunization program for females of eligible age (National Health Commission, September 11, 2025) [25], making a significant step towards improving vaccine accessibility. However, while such policy initiatives are crucial for removing cost barriers, our findings indicate that financial support alone is insufficient, consistent with previous studies [26–28]. Disparities in awareness persist across factors such as rural-urban residence, gender, race, ethnicity, and educational attainment [21–24]. These results highlight the need for targeted educational interventions addressing both structural and perceptual barriers to HPV and HPV vaccination. Furthermore, by examining the role of social media and the emerging gender disparity in trust levels, our study adds a novel dimension to understanding how information sources influence vaccine decision-making in the digital era.

Social media plays a dual role in spreading HPV-related information, functioning both as a major source of awareness and as a potential amplifier of misinformation. In our study, social media was identified as the primary source of HPV-related information for most respondents (77.5%), indicating its broad reach and influence. However, its role is complex. The same platform can increase exposure to health information, while it can also distribute misconceptions and unequal trust across different demographic groups [29,30]. Such dynamics may create substantial disparities in HPV knowledge, as biased or misleading content widens knowledge gaps and disparities, potentially reinforcing vaccine hesitancy. Myths and misinformation have been shown to discourage vaccination uptake among young people [30]. Previous studies in China have reported mixed levels of HPV awareness depending on region, age, and educational background [5,6].

A significant gender disparity in trust towards social media was identified in our study. Male respondents were disproportionately more likely to express distrust in the accuracy of information on these platforms (p = 0.034). This finding aligns with broader concerns about how algorithm-driven content distribution with sex disparities can reinforce biased or misleading health information. Social media’s influence is not neutral but is perceived differently across genders, specifically introducing a disparity in credibility. Strengthening scientific education and health communication is important in the digital age, where social media plays an irreplaceable role in shaping public perception [31,32] and attitudes towards HPV and HPV vaccination.

### 5.1 Implications for public health practice

In light of these challenges, the following strategies are recommended: (i) developing targeted awareness campaigns to address misconceptions among young people; (ii) partnering with digital platforms to develop content-moderation strategies that regulate and improve the quality of HPV-related information on social media; and (iii) advocating government-subsidized vaccination programs to reduce costs barriers.

These practical interventions could help bridge the gaps in HPV knowledge and vaccination intentions identified in our study. Our results suggest that future public health initiatives should move beyond simply raising general awareness and instead focus on correcting specific misconceptions through targeted educational campaigns. Such campaigns should: (i) clearly communicate that HPV is often asymptomatic and emphasize that the vaccine is the prophylactic rather than therapeutic; (ii) collaborate with social media platforms and key opinion leaders to disseminate accurate, evidence-based information and counteract misinformation, with content tailored to address the distrust observed particularly among male users; and (iii) advocate and promote government-subsidized vaccination programs, in line with the recently announced national immunization plan, to reduce the financial barrier, thereby ensuring broader vaccine accessibility for eligible young adults.

### 5.2 Limitations and strengths

The study has several limitations. The relatively small sample size and the use of online sampling method may introduce selection bias, limiting the generalization of the findings. As descriptive study in nature, we did not explore actual vaccination uptake rates or the specific reasons for non-vaccination. Another limitation is the role of digital and social media was not explored, which may influence on gender disparities through algorithm-driven content exposure. While the questionnaire was reviewed and pilot tested, certain dichotomous items may have oversimplified complex concepts, such as the overlapping nature of “safety” and “side effects”. Despite these limitations, the current study provides valuable insights for future HPV education and vaccine promotion efforts in China and beyond. Further larger-scale studies are to be built on these findings and to explore how social media platforms influence public health information dissemination and reshape narratives surrounding HPV and its vaccination.

## 6 Conclusion

The findings highlight misconceptions about HPV’s natural history and vaccine safety remain among even well-educated young adults in China. While overall awareness of HPV is high, and most participants expressed willingness to be vaccinated, knowledge gaps and inconsistent perceptions suggested a lack of fully informed decision-making. These findings show the importance of accessible, culturally tailored education strategies to address specific misunderstanding rather than general hesitancy. Improving the accuracy of HPV-related information-particularly through social media channels-could support public trust and foster more informed vaccine choices.

## 7 Patient and public involvement

Patients and/or the public were not involved in the design, conduct, reporting or dissemination plans of this research.

## Supporting information

S1 Appendix Implied consent and Questionnaire (English version). The document includes the implied consent statement presented to all participants prior to data collection, followed by the complete English (EN) version of the questionnaire.
(PDF)

S2 Appendix Implied consent and Questionnaire (Chinese version). This document provides the complete Chinese (CN) version of the survey instrument, including the implied consent statement and all survey items.
(PDF)

S3 Dataset Raw dataset of HPV knowledge and trust questionnaire responses. This Excel spreadsheet contains the complete, anonymized raw data from the cross-sectional survey, including participant demographics (e.g., age, sex), coded responses to all knowledge and trust questions, and source of information preferences.
(XLSX)
