# Supplementary material for: Bridging knowledge gaps: An observational study on HPV awareness and misconceptions among young adults in China
Source: PLoS One. 2025 Dec 1;20(12):e0337518. doi: 10.1371/journal.pone.0337518 (PMC12668507; doi:10.1371/journal.pone.0337518)
Supplement: S1 Appendix — The document includes the implied consent statement presented to all participants prior to data collection, followed by the complete English (EN) version of the questionnaire. (PDF) [file pone.0337518.s001.pdf]

Bridging Knowledge Gaps: an observational study on HPV awareness and misconceptions among young adults in China

Minrui Li [1¶], Lili Liang [2¶], Xuanyan Chen [3], Zhoujun Zhu [2], Chenyan Fang [4,2], Runan Zhou [3*]

[1] Guangdong University of Finance and Economics, School of Humanities and Communication, Guangzhou, China

[2] Charité-Universitätsmedizin Berlin, corporate member of Freie Universität Berlin and Humboldt-Universität zu Berlin, Department for Gynecology, HPV Research Lab, Augustenburger Platz 1, 13353 Berlin, Germany

[3] Sun Yat-sen University, School of Journalism and Communication, Guangzhou, China

[4] Zhejiang Cancer Hospital, Department of Gynecologic Oncology, Hangzhou, Zhejiang, China

[*] Correspondencing author:

Runan Zhou; zhourn3@mail.sysu.edu.cn

[¶] These authors contributed equally to this work and should be considered co-first authors.

Self-administered HPV associated questionnaire

Implied Consent Statement for the participation

Before this Self-administered questionnaire, please carefully read the following information:

I have fully informed about the study and its purpose. I was approached by the research team in the classroom and given detailed explanations of the purpose and

methodology of the study. I understand that my participation is entirely voluntary and that my responses will remain anonymous and confidential throughout the study. I was given the opportunity to ask additional questions, which were answered to my satisfaction. I have had enough time to consider my participation before giving my consent.

I understand that participation in this study is voluntary and anonymous and that I may withdraw my consent at any time without any reason. I am aware that the data collected will be used only for research purpose, and only authorized researchers will have access to my personal information.

By continuing with the questionnaire, I acknowledge that I have read and understood this information and that I voluntarily agree to participate in the study.

1 Demographic characteristics

Please tick the box to indicate your answer.

1) **What is your Gender?** ☐ Male ☐ Female

2) **Your age is _____?** ☐ Age 18-26 ☐ Age above 26

3) **You are living in ____(Region)**

☐ Mainland ☐ Hong Kong, Macao, Taiwan

4) What is your ethnic group?

☐ Han ☐ Ethnic minorities (please specify) _______________

5) What is your education level?

☐ Bachelor's degree ☐ Master, Doctoral degree or further

6) What is your college major?

☐ Bio-medical associated major (e.g. Medicine, Biotechnology, Pharmacy, etc.)

☐ Non-bio-medical associated major (please specify)

7) Have you ever heard of HPV and HPV infection before?

☐ Yes ☐ No

2 Basic knowledge and awareness of HPV and HPV vaccination

The section was designed to assess your knowledge of HPV and HPV infection. Please answer with： "Yes", "No", or "Don' t know /not sure".

1) HPV is a sexually transmitted virus.
2) HPV is common in China.
3) Both men and women are susceptible to HPV.
4) Regardless of sexual orientation, having unsafe sexual activity increase the risk of HPV infection.
5) Most people who get HPV infection do not have any symptom.

6) HPV can lead to benign genital warts.

7) Persistent HPV infection in women can lead to abnormal cervical dysplasia and/or cervical cancer.

8) HPV may cause other cancers derived from oropharynx, penis and anus among men and women.

9) There is no treatment for HPV infection.

10) There is treatment for HPV induced warts.

11) HPV is associated with infertility.

12) HPV vaccine is effective.

13) It is worth getting HPV vaccine.

14) Vaccination against HPV will prevent HPV-induced cancers, not just cervical cancer.

15) HPV vaccines can adversely affect the immune system.

16) HPV vaccines affect menstrual cycle.

17) Men should also get HPV vaccine.

18) It is too late to get the HPV vaccine after an HPV infection.

3 Health Belief model toward HPV and HPV vaccines using a 5-point (5’) Likert scale

The section included 18 statements categorized into five domains: perceived susceptibility to HPV infection (items 1-4), perceived severity of potential HPV infections (items 5-8), perceived benefits of preventing HPV infection (items 9-12), potential barriers to HPV vaccination (items 13-15), and self-efficacy related to receiving HPV vaccination (items 16-18).

Your agreement with each statement should be rated on a 5-point Likert scale. Please choose the option that best corresponds with your feelings about each statement, rated on a scale where 1’ = Strongly disagree, 2’ = Disagree, 3’ = Neither agree nor disagree (Neutral), 4 ‘= Agree, and 5 ‘= Strongly agree.

1) Anyone who is sexually active is at risk of HPV infection.
2) Many people can be infected with HPV, including my relatives, partners, and friends.
3) I may be infected with HPV in the future.
4) I am at high risk for HPV infection.
5) HPV infection is terrible.
6) HPV infection can lead to serious health problem, threatening your wellbeing.
7) HPV infection can seriously affect your daily life.
8) HPV infection can cause death.
9) HPV vaccination can benefit me.
10) HPV vaccination can help my immune system fighting against HPV.
11) HPV vaccination can reduce the risk of HPV infection.
12) HPV vaccination can make sexual activity safer.
13) I am concerned about the safety of the HPV vaccine.
14) I am concerned about the potential side effects of the HPV vaccine.
15) I am worried that getting the HPV vaccine will take up too much of my time and effort
16) I would consider getting the HPV vaccine.
17) I am going to get the HPV vaccine.
18) I will get the HPV vaccine.

4 Concerns about the HPV vaccine

The section of concerns about the HPV vaccine is to predict your willingness to receive HPV vaccine, please answer with “yes” or “no”.

1) Are potential side effects your greatest concern about HPV vaccine?

2) Is cost your greatest concern about HPV vaccine?

3) Is the effectiveness and safety of the vaccine your greatest concern about HPV vaccine?

4) Is any other concern not listed here your greatest concern about HPV vaccine?

5 Opinions, Attitudes, and Behaviors Regarding HPV Vaccination

This section aims to understand your views and stance on HPV vaccination. Please select the option that best reflects your opinion.

1) Who should receive the HPV vaccine? (Select one only)

☐ Males only ☐ Females only

☐ Both males and females ☐ Not sure

2) When do you think is the appropriate time to receive the HPV vaccine? (Select one only)

☐ Before 9 years old ☐ During adolescence

☐ At any age ☐ Before first sexual activity

☐ Before marriage ☐ Not sure

3) If a healthcare professional recommends the HPV vaccine, would you be willing to receive it?

☐ Yes ☐ No ☐ Not sure

4) Do you think it is too late to get vaccinated against HPV after already being infected with the virus?

☐ Yes ☐ No ☐ Not sure

5) Which type of HPV vaccine do you think should be chosen? (Select one only)

☐ Bivalent ☐ Quadrivalent

☐ Nonavalent ☐ Any type, as long as an appointment can be made

6) Which do you believe is more important regarding HPV vaccination? (Select one only)

☐ Timing (the earlier, the better) ☐ Valency of the vaccine

7) Who do you think should make the decision about whether you receive the HPV vaccine? (Select one only)

☐ Yourself ☐ A joint decision between you and your parents

◻ Your parents' decision ◻ Not sure

6 Sources and Trust of HPV-Related Health Information

This section aims to understand how you access and perceive information related to HPV. Please select the option that best reflects your situation.

1) How do you obtain information about HPV? (Select all that apply)

□ Healthcare professionals or medical institutions ☐ Social media

□ Friends or family members ☐ School or health education courses

2) How often do you browse information about HPV and/or HPV vaccines on social media? (Select one only)

□ Never ☐ Rarely ☐ Occasionally

☐ Frequently ☐ Always

3) How much do you trust the information about HPV and/or HPV vaccines that you receive from social media? (Note: social media refers to services that use computers or the Internet to provide information or entertainment.) (Select one only)

□ Strongly trust ☐ Somewhat trust ☐ Neutral

□ Somewhat distrust ☐ Strongly distrust

The questionnaire ends here.

Thank you very much for completing the questionnaire. Please check that you have answered all the questions. Thank you again for your time and effort. Your participation is greatly appreciated.
