# Supplementary material for: Bridging knowledge gaps: An observational study on HPV awareness and misconceptions among young adults in China
Source: PLoS One. 2025 Dec 1;20(12):e0337518. doi: 10.1371/journal.pone.0337518 (PMC12668507; doi:10.1371/journal.pone.0337518)
Supplement: S2 Appendix — This document provides the complete Chinese (CN) version of the survey instrument, including the implied consent statement and all survey items. (PDF) [file pone.0337518.s002.pdf]

Bridging Knowledge Gaps: an observational study on HPV awareness and misconceptions among young adults in China

Minrui Li [1¶], Lili Liang [2¶], Xuanyan Chen [3], Zhoujun Zhu [2], Chenyan Fang [4,2], Runan Zhou [3*]

[1] Guangdong University of Finance and Economics, School of Humanities and Communication, Guangzhou, China

[2] Charité-Universitätsmedizin Berlin, corporate member of Freie Universität Berlin and Humboldt-Universität zu Berlin, Department for Gynecology, HPV Research Lab, Augustenburger Platz 1, 13353 Berlin, Germany

[3] Sun Yat-sen University, School of Journalism and Communication, Guangzhou, China

[4] Zhejiang Cancer Hospital, Department of Gynecologic Oncology, Hangzhou, Zhejiang, China

[*] **Correspondencing author:**

Runan Zhou; zhourn3@mail.sysu.edu.cn

[¶] **These authors contributed equally to this work and should be considered co-first authors.**

关于人乳头瘤病毒（HPV）的在线调查问卷（中文版）

HPV online Questionnaire (CN version)

知情同意书 (Implied consent)：参与关于 HPV 和疫苗接种的认识的调查问卷

在进行这个自填式的在线调查问卷前，请您仔细阅读以下信息：

我已充分了解本研究及其目的。研究人员在教室与我接触，并详细说明了研究目的及方法。我理解参与完全基于自愿原则，且我的所有回答在整个研究过程中将保持匿名和保密。

我有权提出额外问题，并已得到满意解答。在作出参与决定前，我已获得充分时间进行考虑。

我知悉参与本研究属自愿行为且采取匿名方式，可随时无理由撤回同意。我了解所收集数据仅用于科研目的，仅授权研究人员可接触我的个人信息。

通过继续填写问卷，我确认已阅读并理解上述内容，并自愿同意参与本研究。

导言

人乳头瘤病毒（HPV）有 100 多个变种。HPV 病毒引起的感染通常会导致皮肤或粘膜病灶。某些类型的人乳头瘤病毒 (HPV) 感染会导致疣 （例如， 尖锐湿疣），而另一些则会导致不同类型的癌症。

人乳头瘤病毒疫苗（HPV vaccine）是预防某些特定类型人乳头瘤病毒感染的疫苗。 现有的 HPV 疫苗可预防两种、四种或九种类型的 HPV。 所有 HPV 疫苗至少可预防 HPV 16 和 18 型，这两种类型导致 HPV 相关癌症的风险最大。

感谢您参与本次调查问卷。

您的反馈对我们来说非常宝贵。 请花几分钟时间完成以下问题。

第 1 章 个人信息 demographic characteristics

请阅读以下问题，并勾选您的答案。

1. 性别: ☐ 男 ☐ 女

2. 年龄： ☐ 18-26 岁 ☐ >26 岁

3. 所在地区：☐ 内陆 ☐ 港澳台

4. 民族： ☐ 汉族 ☐ 少数民族（请具体说明）_______________

5. 教育程度 Education level： ☐ 本科 ☐ 硕博及以上

6. 专业 College major：

☐ 生物医学相关专业（如医学、生物技术、药学等）

☐ 非生物医学相关专业（请具体说明）_______________

7. 您之前是否听说过 HPV 感染？ ☐ 是 ☐ 否

第 2 章 HPV 与 HPV 疫苗接种基础知识认知

本部分旨在评估您对 HPV 及其感染的认知情况。请选择“是”、“否”或“不知道/不确定”。

1. HPV 是一种通过性传播的病毒。
2. HPV 在中国较为常见。
3. 男性和女性都有可能感染 HPV。
4. 无论异性恋还是同性恋，不安全的性行为都会增加感染 HPV 的风险。
5. 大多数 HPV 感染者没有明显症状。
6. HPV 可能引起生殖器疣，例如尖锐湿疣。
7. 如果女性持续存在 HPV 感染，可能导致宫颈异常病灶和/或宫颈癌。
8. 除子宫颈癌外，HPV 还可能导致男性和女性患上其他癌症，例如口咽癌、阴茎癌和肛门癌。
9. 目前还没有针对 HPV 病毒的有效治疗办法。
10. HPV 引起的疣体可治疗。
11. HPV 感染与不孕有关。
12. PV 疫苗是有效的预防手段。
13. 接种 HPV 疫苗是值得的。
14. 接种 HPV 疫苗可以预防 HPV 相关的癌症，不仅仅限于预防宫颈癌。
15. HPV 疫苗会对免疫系统产生不利影响。
16. HPV 疫苗会影响月经周期。
17. 男性也应该接种 HPV 疫苗。

18. HPV 感染后再接种 HPV 疫苗为时已晚。

第 3 章 HPV 和 HPV 疫苗相关的健康信念模型 Health Belief model（HBM）

本部分共包含 18 项陈述，分为五个维度：HPV 感染感知易感性（第 1-4 题）、HPV 感染感知严重性（第 5-8 题）、HPV 预防感知收益（第 9-12 题）、疫苗接种潜在障碍（第 13-15 题）以及疫苗接种自我效能（第 16-18 题）。

请根据您对每项陈述的认同程度，选择最符合您真实感受的选项。评分标准如下：1=非常不同意，2=不同意，3=中立，4=同意，5=非常同意。

1. 任何有性行为者均面临 HPV 感染风险。
2. 包括亲友、伴侣在内的许多人均可能感染 HPV。
3. 我未来可能感染 HPV。
4. 我属于 HPV 感染高风险人群。
5. HPV 感染后果严重。
6. HPV 感染会引发威胁健康的重大疾病。
7. HPV 感染会严重影响日常生活。
8. HPV 感染可能导致死亡。
9. 接种 HPV 疫苗对我有益。
10. HPV 疫苗能增强免疫系统对 HPV 的抵抗能力。
11. HPV 疫苗可降低感染风险。
12. HPV 疫苗能使性行为更安全。
13. 我担心 HPV 疫苗的安全性。
14. 我担心 HPV 疫苗可能产生的副作用。
15. 我担心接种 HPV 疫苗会耗费过多时间与精力。
16. 我会考虑接种 HPV 疫苗。

17. 我已有计划去接种 HPV 疫苗。

18. 我准备接种 HPV 疫苗了。

第 4 章 旨在调查参与者对 HPV 疫苗的顾虑

本部分旨在了解您对接种 HPV 疫苗可能存在的顾虑。请根据您的实际情况，选择“是”或“否”。

1. 潜在副作用是否是您对 HPV 疫苗的最大顾虑? ☐ 是 ☐ 否
2. 接种费用是否是您对 HPV 疫苗的最大顾虑? ☐ 是 ☐ 否
3. 疫苗的有效性与安全性是否是您对 HPV 疫苗的最大顾虑? ☐ 是 ☐ 否
4. 是否存在未列出的其他因素构成您对 HPV 疫苗的最大顾虑? ☐ 是 ☐ 否

第 5 章 HPV 相关健康信息的来源与信任度调查

1. 您如何获取 HPV 相关知识的, 途径有哪些? （单选/多选）

   ☐ 医生或医疗机构 ☐ 社交媒体

   ☐ 朋友或家人的分享 ☐ 学校或健康教育的课程

2. 您通过社交媒体浏览 HPV/HPV 疫苗相关信息的频率是? （单选）

   ☐从不 ☐很少 ☐有时

   ☐经常 ☐总是

3. 您对社交媒体中 HPV/HPV 疫苗信息的信任程度如何? （注：社交媒体指通过计算机或互联网提供信息及服务的媒介）（单选）

   ☐非常信任 ☐比较信任 ☐一般

   ☐比较不信任 ☐非常不信任

（至此问卷结束。）

衷心感谢您完成本问卷。

请确认已回答所有问题。

再次感谢您付出的时间与精力，您的参与对我们具有重要意义。
